# Impact of Short-Term HAART Initiated during the Chronic Stage or Shortly Post-Exposure on SIV Infection of Male Genital Organs

**DOI:** 10.1371/journal.pone.0037348

**Published:** 2012-05-17

**Authors:** Marina Moreau, Anna Le Tortorec, Claire Deleage, Charles Brown, Hélène Denis, Anne-Pascale Satie, Olivier Bourry, Nathalie Deureuddre-Bosquet, Pierre Roques, Roger Le Grand, Nathalie Dejucq-Rainsford

**Affiliations:** 1 INSERM U1085-IRSET, Université de Rennes 1, Institut Fédératif de Recherche 140, Rennes, France; 2 CEA, Division of Immunovirology, Institute of Emerging Diseases and Innovative Therapies, DSV, Fontenay-aux-Roses, France; 3 Laboratory of Molecular Microbiology, National Institute of Allergy and Infections Diseases, National Institutes of Health, Bethesda, Maryland, United States of America; 4 UMR-E1, Université Paris-Sud 11, Orsay, France; Institut Pasteur, France

## Abstract

**Background:**

The male genital tract is suspected to constitute a viral sanctuary as persistent HIV shedding is found in the semen of a subset of HIV-infected men receiving effective antiretroviral therapy (HAART). The origin of this persistent shedding is currently unknown. Phylogenetic studies indicated that HIV in semen from untreated men arises from local sources and/or passive diffusion from the blood. We previously demonstrated in human and macaque low levels and localized infection of several semen-producing organs by HIV/SIV. Using a macaque model, this study investigates the impact of short term HAART (2–4 weeks) initiated either during the asymptomatic chronic stage or 4 h post-intravenous inoculation of SIVmac251 on the infection of male genital organs.

**Methodology/Principal Findings:**

Short term HAART during the chronic stage decreased blood viral load. No major impact of HAART was observed on SIV DNA levels in male genital organs using a sensitive nested PCR assay. Using *in situ* hybridization, SIV RNA+ cells were detected in all male genital tract organs from untreated and treated animals with undetectable blood viral load following HAART. Infected CD68+ myeloid cells and CD3+ T lymphocytes were detected pre- and post-HAART. In contrast, short term HAART initiated 4 h post-SIV exposure led to a drastic decrease of the male genital tissues infection, although it failed to prevent systemic infection. In both cases, HAART tended to decrease the number of CD3+ T cells in the male organs.

**Conclusions:**

Our results indicate that the established infection of male genital organs is not greatly impacted by short term HAART, whereas the same treatment during pre-acute phase of the infection efficiently impairs viral dissemination to the male genital tract. Further investigations are now needed to determine whether infection of male genital organs is responsible for long term persistent HIV shedding in semen despite HAART.

## Introduction

Highly active antiretroviral therapy (HAART) significantly improved the clinical outcome among HIV-infected patients, leading in most patients to undetectable viremia (i.e. <50 copies/ml). Nonetheless, viral eradication is not achieved as HIV continues to replicate at low level in several tissues or remains under a latent form in several cellular and anatomical sites, called viral reservoirs. The male genital tract (MGT) is suspected to constitute such a pharmacological sanctuary or reservoir. Indeed persistent shedding of HIV RNA and infectious particles is detected in the semen of a subset of chronically-infected men under prolonged effective HAART [1], [2], [3], [4], [5], [6] (other references in [7]). In some of those men, the seminal viral load can reach several log10 of magnitude, despite undetectable virus in blood for years [1], [3], [4], [5], [7]. The origin of this persistent shedding is currently unknown. It does not appear to correlate to suboptimal drug concentrations in semen nor to any specific drug regimen, and occurs in the absence of other detectable sexually transmitted infections, known to increase the release of HIV in semen [7]. High seminal viral load before treatment initiation is to date the only factor found to correlate with persistent release of HIV in semen despite HAART [3]. Several phylogenetic studies demonstrated that HIV in semen arises from local productive sources within the MGT and/or, depending on the individuals, from passive diffusion from the blood [8], [9], [10] (previous references in [7]). Compartmentalization of seminal strains was also recently shown in macaques [11]. We and others have revealed that several MGT organs are infected by HIV/SIV during the primary and asymptomatic chronic stages of the infection [12], [13], [14], [15], [16], [17], [18]. Productive infection was detected in the prostate, seminal vesicle, epididymis, and to a lower level in the testis [12], [13], [14], [15], [16], [17], [18]. The latter represents a well known pharmacological sanctuary for many drugs, including antiretrovirals against HIV [19], [20]. All these elements strongly suggest that one or several MGT organs constitute a viral sanctuary that can keep producing virus despite HAART, and contribute to the discordant blood/semen viral loads observed in some patients under HAART.

Although the importance of tissue sanctuaries is being increasingly recognized [21], the impact of HAART on semen-producing organs has never been thoroughly investigated. The lack of access to genital organ material from men receiving HAART as well as the difficulties in detecting infection of the MGT organs *in vivo* due to the low level and localized nature of this infection [12], [14], have impaired research in this domain. Using the simian immunodeficiency virus (SIV)-infected cynomolgus macaque model and as a first step in testing the impact of HAART on MGT organs infection, this study examine the effect of short term HAART administrated for 2 to 4 weeks during the asymptomatic chronic stage on the infection of testis, epididymis, prostate and seminal vesicle. In addition we investigated the impact of HAART initiated very shortly post-infection, i.e. 4 h post intravenous inoculation, an early time we previously demonstrated does not prevent systemic infection [21], [22].

## Methods

### Animals and Ethics Statement

20 adult male cynomolgus macaques (*Macaca fascicularis*) (3–4 years old, body weight >5 Kg, all mature as attested by the presence of full spermatogenesis) were imported from Mauritius Island and housed in the facilities of the Centre d'Energie Atomique (CEA) (Fontenay-aux-Roses, France). Non-human primates (NHP) are used at the CEA in accordance with French national regulation and CEA facilities are fully authorized under the number B-92-032-02 for animal use and under the number 2005-69 for NHP breeding. These facilities are regularly inspected by national veterinary inspectors. The CEA is in compliance with ETS123 recommandations, Directive 86/609/CEE guidelines (Directive 2010/63/CE guidelines entering in force in 2013 are also implemented) for animal breeding and with Standards for Human Care and Use of Laboratory Animals (Animal Welfare Assurance, OLAW number #A5826-01). NHP are daily fed and inspected by animal caretakers who report directly to the veterinarians in charge of the animal facilities and animal welfare. The head of the veterinarian staff is empowered to interrupt the protocol in case of animal pathology or suffering. It should be stressed that none of the animal were specifically used for this work, since the male genital organs were collected at necropsy from animals that were euthanized in the course of other studies [22] thus no suffering was specifically associated with the surgical procedure to obtain the male genital organs. This approach is fully in accordance with the 3R and reduces the number of animal used as recommended by the Directive 2010/63/CE (article 18 sharing tissues and organs). Animal suffering avoidance and refinement of procedures are included in CEA aims. The use of NHP at CEA is in accordance with recommendation of the Weatherall report as follows: NHP are used at CEA only when no other models (*in vitro* or *in vivo*) are suitable for the aims of the project (recommendation n°1); the study is in the field of infectious diseases (recommendation n°2); NHP are breed following the recommendation of the ETS123 and in accordance with the newly published European Directive (2010/63) (recommendation n°9). The animals were used under the supervision of the veterinarians in charge of the animal facility and the protocols employed were reviewed by the Ethical Animal Committee of the CEA (Ethical Animal Committee registered by French national under the number 44). The protocols and the use of male genital organs for the purpose of the work described herein were approved by the Ethical Animal Committee of the CEA under the number 11-005. The animals were sedated with ketamine chlorydrate (Rhone-Merieux, Lyon, France), before virus injection, blood sample collection, and before receiving treatment, as previously described [23]. Tissues from the MGT were collected during animal necropsy after sedation of animals (ketamine chlorhydrate 10 mk/kg) followed by euthanasia (sodium pentobarbital 180 mg/kg).

### Infection and treatment

The animals were intravenously inoculated with 50 AID_50_ of pathogenic cell-free SIVmac251 as previously described [23]. Ten macaques (16746, 20475, 20785, 20828, 20929, 20972, 9770, 10010, 10015, 10505) were given twice a day two nucleoside inhibitors, zidovudine (AZT, 4.5 mg/kg) and lamivudine (3TC, 2.5 mg/kg) subcutaneously, and the protease inhibitor indinavir (IDV, 60 mg/kg) orally, whilst ten macaques were untreated (8141, 9345, OBCB5, OBCJ5, 20351, 30675, 9368, 9680, 10092, 10466). Treatment was initiated 4 h (for animals 9770, 10010, 10015, 10505) or 21 weeks post-inoculation (p.i.) (for animals 16746, 20475, 20785, 20828, 20929, 20972) and was continued for 2 to 4 weeks. The 4 week time point was designed to allow reducing systemic infection. Intermediate time point at 2 week of treatment was also explored. Animals were euthanized at the end of the treatment.

### Specimen collections and blood viral load measurement

Plasma viral loads (PVLs) and PBMC-associated viral loads were assessed at several time points as previously described [24], [25]. Tissues from the MGT (testis, epididymis, prostate and seminal vesicle) were collected immediately after euthanasia and exsanguination of the animals, extensively washed and cut into fragments weighing about 300 mg each. The fragments were either stored at −80°C or fixed in 4% formaldehyde.

### Nucleic acids extraction

Total DNA and RNA were extracted from 2 distinct fragments of each tissue using RNeasy isolation kit or the QIAamp DNA Blood mini kit (both Qiagen, Courtaboeuf, France), respectively. RNA samples were depleted of contaminating DNA by DNase treatment (Promega, Charbonnières, France) and submitted to reverse transcriptase reactions, using random hexamer primers (Boehringer-Mannheim, Mannheim, Germany) and M-MLV-Reverse Transcriptase (Invitrogen, Cergy-Pontoise, France) to generate cDNA. Total DNA from PBMCs was isolated using a commercial kit (Genomic DNA from tissue, Macherey-Nagel, GmbH & KG, Germany).

### Nested PCR for SIV DNA and RNA

A previously described sensitive nested PCR [14], [26] was used to detect SIV gag DNA. In order to increase the chances of detection of focal infection of the genital tissues, 2 independent fragments of each tissue were assayed in a minimum of 18 PCR reactions, each performed on 500 ng of extracted DNA, as we previously described [14]. The sensitivity was 100% for the detection of 10 copies of SIVmac251 gag DNA in 500 ng of exogenous DNA, and 33% for a detection threshold of 1 copy. The lack of contamination in PCR assays was systematically ensured by concurrently running a minimum of 6 negative controls. Results were expressed as percentages of SIV gag DNA positive PCR. The presence of SIV tat-rev mRNA was analyzed using a nested RT-PCR protocol adapted from Pasternak *et al* [27]. 40 cycles of first round PCR amplification were performed using primers specific for the tat-rev mRNA chosen based on [27] (TatrevF1 5′:CCTCCTCCAGGACTTGCATA and TatrevR1 5′: CTGTTGATGACTGCCCGATA). A second nested PCR amplification (40 cycles) was performed with 3 µL amplimer and inner primers TatrevF2 5′:GCAGCAATCCATATCCACAG and TatrevR25′: GAGGGTCAGGCAGATGTTGT.

### Immunohistochemistry

The following human mAbs and matching isotype controls were used at the indicated concentrations: anti-HLA-DR (TAL.1B5, 0.6 µg/ml, a marker of activated immune cells), anti-CD68 (KP1, 1.2 µg/ml, a marker of myeloid cells), anti-CD3 (F7.2.38, 6.75 µg/ml, a marker of T lymphocytes) (all from Dako SA, Trappes, France), anti-CD4 (1F6, Novocastra, 2.5 mg/ml), anti-TIA-1 (2G9, Immunotech, 1 mg/ml, a marker of cytotoxic cells), with mouse IgG1 isotype control (Dako); anti-CD20 (L26, Dako, 0.44 mg/ml, a marker of B lymphocytes), with mouse IgG2a control (BD Biosciences). Immunohistochemistry was performed as previously described [16]. No staining was ever observed with isotype control antibodies. A minimum of three sections from distinct areas were observed per animal. For quantitative and semi-quantitative measurement, cell counts were performed using the Cast software (Olympus) on a minimum of two sections per animal. For CD3+T cell infiltrates semi-quantitative analyses, whole sections were examined.

### In situ hybridization (ISH)

Formalin-fixed, paraffin-embedded tissues were assayed for SIV RNA expression by radioactive and non radioactive ISH, using previously described ^35^S-UTP-labeled riboprobe for the gag region of SIVmac251 [14] or digoxigenin-UTP-labeled riboprobe spanning the whole genome of SIVmac239 [28]. Radioactive ISH was performed as we previously described [14], [16]. Non radioactive ISH experiments were performed according to published procedures [29], [30], with the modifications indicated below. For localization and semi-quantitative analysis of infected cells in the tissues, nitroblue tetrazolium-5-bromo-4-chloro-3-indollylphosphate toluidinium (NBT-BCIP) revelation was used [29]. Briefly, the sections were deparaffinized, pretreated with proteinase K, and hybridized overnight at 45°C with either sense or antisense SIVmac239 digoxigenin-UTP-labeled riboprobe (Lofstrand Labs, Gaithersburg, MD, USA). The hybridized sections were washed with posthybridization buffers and RNase solutions and blocked with Protein block serum-free (Dako), before incubation with sheep anti-digoxigenin-alkaline phosphatase (Roche Molecular Biochemicals) for 1 h at room temperature. The sections were then reacted with NBT/BCIP for 12 h at room temperature, rinsed with distilled water and counterstained with Fast red (Sigma-Aldrich). The specificity of the hybridization signal was systematically checked by hybridizing sense probes on parallel sections and anti-sense probes on uninfected genital tissues. SIV RNA positive cells were counted for two animals/group in a minimum of 30 adjacent tissue sections/experiment, in two independent experiments performed on distinct tissue blocks. The total surface area counted was determined using the Cast Grid software (Olympus, France). Characterization of infected cells was performed using either radioactive or non radioactive ISH combined with immunostaining for cell markers, as described before [16], [30]. Briefly, for radioactive ISH method, the sections were incubated either with a mouse monoclonal antibody against human myeloid cell antigen CD68 (clone KP1, Dako, 1.57 µg/ml) before the ISH or with a mouse monoclonal antibody against human CD3 antigen (F7.2.38, Dako, 6.75 µg/ml) after the ISH. The staining was visualized using diaminobenzidine substrate (Dako). For non radioactive ISH, sections were treated with methanol-hydrogen peroxide, and the hybridized tissues incubated with sheep anti-digoxigenin peroxydase (SAD-POD, Roche Molecular Biochemicals) for 1 h at room temperature. SAD-POD was detected by a fluorescent tyramide signal amplification technique (TSA Plus FITC, NEL741; Perkin Elmer). Following ISH assay, the sections were incubated either with a mouse monoclonal antibody against human myeloid cell antigen CD68 (KP1, Dako, 3.6 µg/ml) and subsequently stained with a goat Alexa-594 anti-mouse IgG antibody (4 µg/ml, Invitrogen BP, Cergy-Pontoise, France), or with a polyclonal rabbit anti-human CD3 antibody (A0452, Dako, 6.7 µg/ml) and revealed using goat Alexa-594 anti-rabbit IgG antibody (4 µg/ml, Invitrogen BP, Cergy-Pontoise, France). The double stained sections were mounted in Vectashield mounting medium with Dapi (Vectashield, Vector Laboratories, Ltd., Peterborough, England).

### Statistical analyses

The significance of the differences between values was evaluated using the appropriate non parametric test, as specified in the text or the figure legends. P<0.05 was considered statistically significant. Statistical analyses were performed using commercially available software (SAS version 9.1.3; SAS Institute, Inc., Cary, NC).

## Results

### Effect of short term HAART initiated during early chronic stage

**Figure 1:**
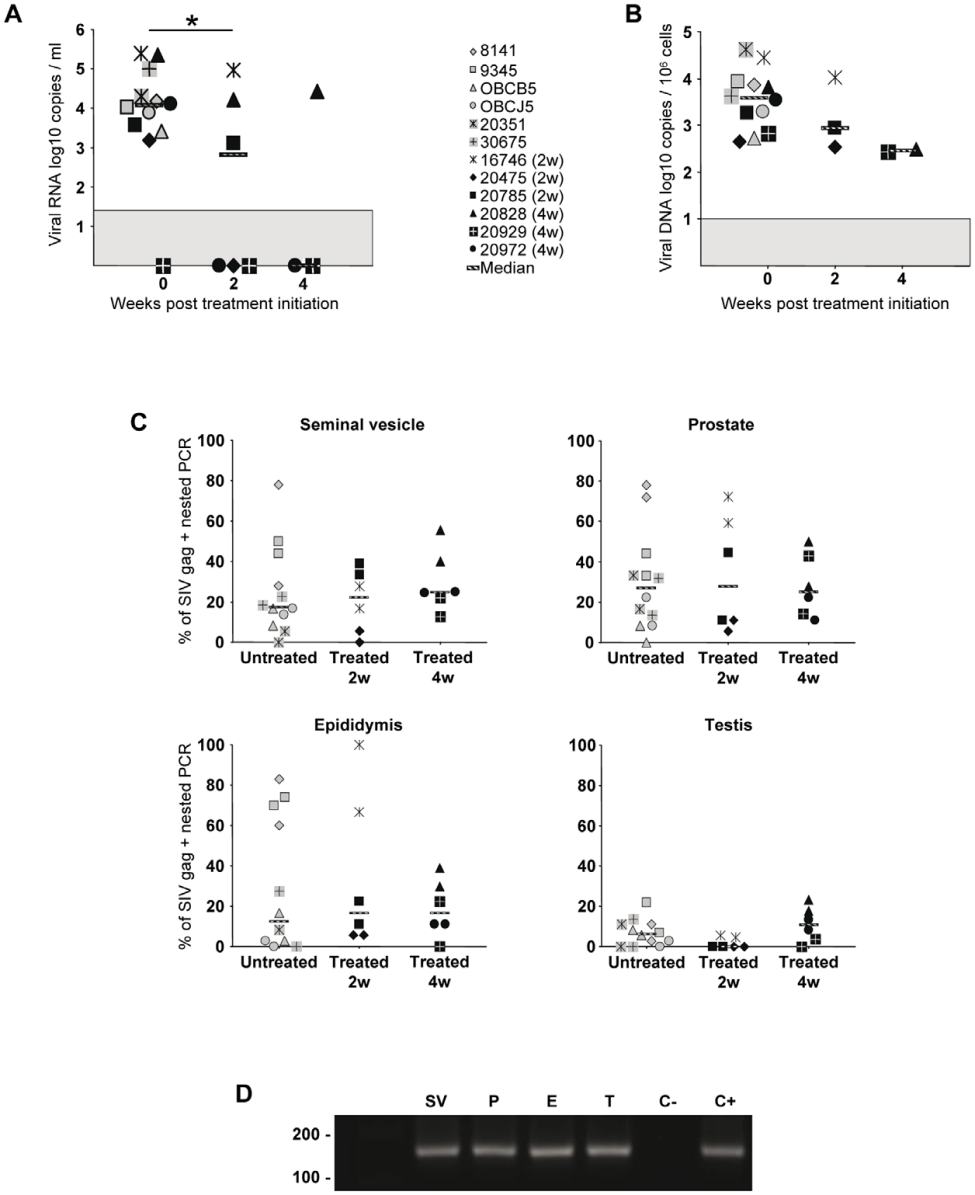
Viral dynamics in chronically-infected macaques receiving HAART and SIV nucleic acids detection in MGT organs. 6 animals were untreated, 3 received a 2 weeks long AZT/3TC/IDV treatment and 3 received 4 weeks of the same treatment. (A) Plasma viral load and (B) total viral DNA in PBMCs were measured before and after treatment. Median is represented by a line. The grey area indicates the quantitative threshold of the qRT-PCR and qPCR assays. Star indicates statistical differences (p<0.05) using a Wilcoxon test. (C) Frequency of detection of SIV DNA in the seminal vesicle, prostate, epididymis and testis of untreated, 2 or 4 week treated macaques, using nested SIV gag PCR. Two independent fragments of each tissue were assayed. Median of frequency is represented by a line. (D) Tat/rev spliced RNA detection in MGT organs (SV: seminal vesicle; P: prostate; E: epididymis, T: testis) by RT nested PCR. Results are shown for one representative treated animal (20475) with an undetectable PVL after treatment. C− and C+ represent negative and positive controls respectively.

The pharmacokinetics of the three antiretroviral drugs used in the present study were previously assessed in blood plasma and PBMCs from cynomolgus macaques [24] and the virological efficacy of the treatment demonstrated in the same animals than the ones used in this study [22]. Before treatment, the median for blood viral load for the 12 animals studied was 4 log10 (Figure 1A). Six animals were treated with AZT/3TC/IDV twice a day after viral set point (from day 150 pi). To determine the kinetics of viral load decrease in tissues, three of these animals were euthanized 2 weeks, and the other three 4 weeks, after the onset of treatment. Among the HAART treated macaques, one (20929) had undetectable PVL at the initiation of therapy but was nevertheless included in the analysis since its PBMC-associated viral load evolved within the same range as the other treated macaques (Figure 1B). HAART for 2 to 4 weeks induced a decreased PVL in the 5 out of 5 animals with a detectable PVL before treatment (Figure 1A). This decrease was significant after 2 weeks (p = 0.0139, Wilcoxon). At the end of the treatment, three animals (20475, 20972 and 20929) out of 6 had PVL below detection threshold (60 vRNA copies/ml). The median PVL for the animals sacrificed at 2 and 4 weeks post-HAART were respectively 3.11 log10 and below detection threshold. As previously observed in HIV-infected patients, SIV DNA in PBMCs was only slightly reduced by treatment, with a median reduction of 0.6 log10 after HAART for 2 weeks and 1.1 log10 after HAART for 4 weeks, as compared with animals without treatment (Figure 1B). We previously showed that SIV DNA in MGT organs of chronically infected macaques can only be detected using a sensitive nested PCR for SIV gag DNA, as infection levels are below the limit of detection of quantitative SIV PCR assays [14]. In untreated animals, the median frequency of viral DNA detection was lower in the testis (6.2%) compared with the prostate, seminal vesicle and epididymis (27, 17.4 and 12.4% respectively) (Figure 1C). The frequency of viral DNA detection per tissue was relatively heterogeneous among animals, and sometimes in between two tissue fragments of the same animal, suggesting localized infection of the tissue. No major impact of HAART was observed on SIV DNA median frequency of detection in the MGT organs of the treated animals for 2 or 4 weeks, as compared with the untreated group (Figure 1C). In order to search for active viral replication into MGT tissues, the presence of spliced tat/rev mRNA was analyzed by nested RT-PCR. Tat/rev mRNA could be detected in all tissues from treated animals, even those with undetectable PVL following HAART (Figure 1D).

**Figure 2:**
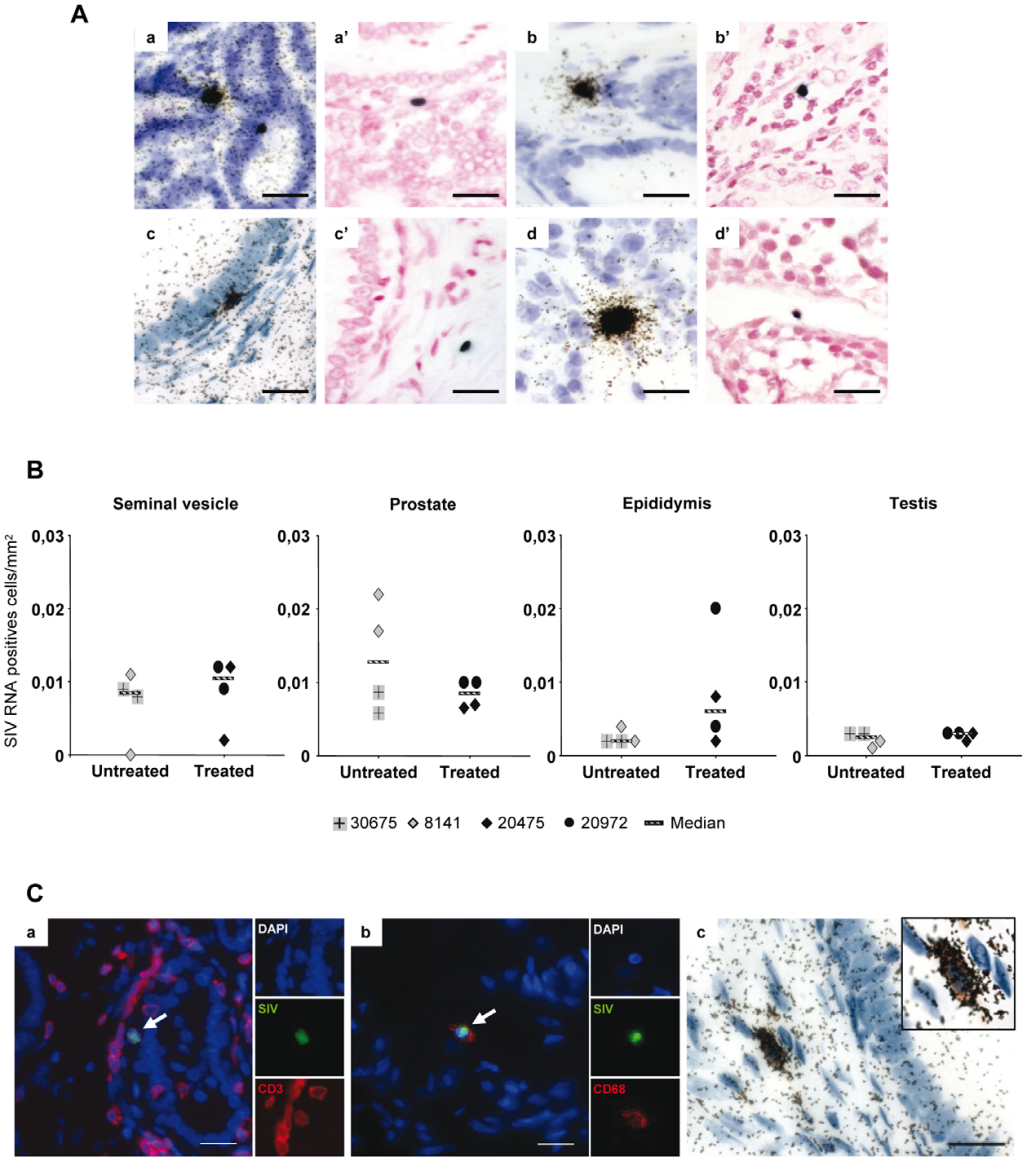
SIV RNA+ cells detection and identification within MGT of chronically infected macaques treated or not. (A) Detection of SIV positive cells in the seminal vesicle (a, a′), prostate (b, b′), epididymis (c, c′) and testis (d, d′) of macaques, treated or not, using radioactive (a, b, c, d) or non-radioactive (a′, b′, c′, d′) ISH for SIV gag RNA. Scale bars = 20 µm. (B) Due to the low and localized nature of the infection, an extensive screening for SIV RNA+ cells was performed in a minimum of 30 tissues section/experiment in 2 independent radioactive ISH experiments on the seminal vesicle, prostate, epididymis and testis of 2 untreated and 2 treated macaques with undetectable PVL following HAART. Median is represented by a line. (C) Identification of SIV-expressing cells using either non-radioactive (a, b) or radioactive (c) ISH for SIV RNA combined with immunostaining for cell markers in treated versus untreated animals. The pictures presented show the detection of CD3+ (a, red) or CD68 (b, red) SIV+ (green) co-labeled cell in the prostate (a) and epididymis (b) of one treated animal. Nuclei labeled with DAPI are shown in blue. Side panels represent individual channels. Large panel represents a merged image combining all channels. Arrows show co-labeled cells. (c) Detection of CD68+ (brown) SIV+ (black dots) co-labeled cell in the epididymis of one treated animal with undetectable PVL following HAART. Insert show enlargement of co-labeled cell. Scale bars = 20 µm.

*In situ* hybridization for SIV gag RNA using either ^35^S or digoxygenin labeled riboprobes revealed positive cells in all the MGT tissues from both untreated and treated animals (Figure 2A). The sensitivity of detection appeared similar between the two techniques. Due to the low number and localized distribution of infected cells, an extensive screening of large tissue areas (on average 3315 mm^2^/tissue/animal, minimum of 1026 mm^2^) had to be undertaken for all the male genital organs in order to measure the number of SIV+ cells. In accordance with the findings on viral DNA and with our previous findings on primary-infected macaques [14], the testis of the 2 untreated animals tested systematically displayed a lower number of infected cells compared with the other MGT organs (Figure 2B). Overall, the number of SIV RNA+ cells observed in MGT tissues from both treated (n = 2) and untreated animals (n = 2) was low and heterogeneous within the same organ of the same animal, hampering precise quantification. This was particularly true for the epididymis and seminal vesicle, which displayed very localized infection. Overall, the analysis of the number of infected cells in the MGT organs of treated macaques with undetectable PVL following HAART compared with that of untreated animals with similar PVL before HAART did not reveal any major impact of HAART in MGT tissues (Figure 2B).

To determine the nature of the infected cells, co-localization of SIV RNA+ cells with cell markers (CD3 for T lymphocytes and CD68 for myeloid cells) was performed. A mix of CD3+ and CD68+ infected cells was observed in MGT tissues from untreated animals, as we previously described [14]. In treated animals, both SIV+CD68+ and SIV+CD3+ cells were encountered in the male genital tract using either radioactive or non radioactive ISH (Figure 2C).

### Impact of short term HAART on immune infiltrates in MGT organs of chronically infected animals

**Figure 3:**
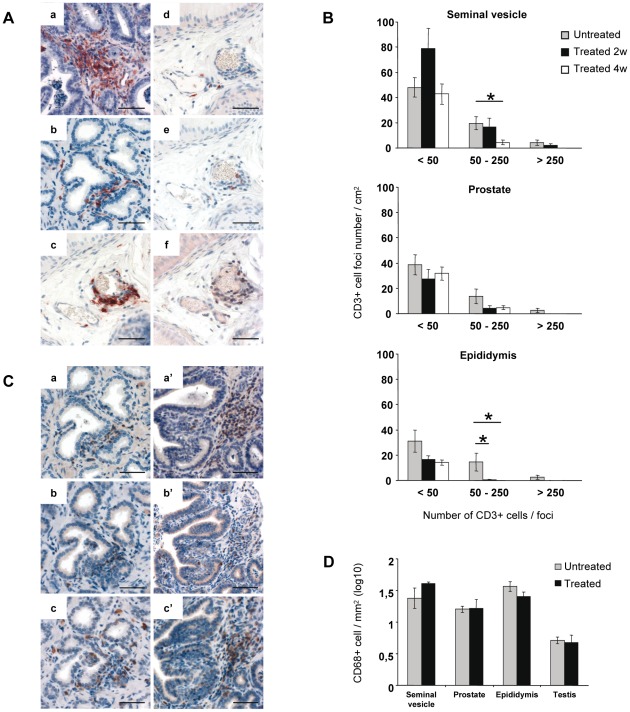
Inflammatory infiltrates in the MGT organs of chronically infected macaques treated or not. (A) Immunohistochemical staining of tissue sections from a treated animal with undetectable PVL after treatment. Detection of HLA-DR+ cells in the seminal vesicle (a), prostate (b) and epididymis (c). Serial tissue sections were stained with anti-CD68 (d), anti-CD20 (e) and anti-CD3 antibodies (f) (photos shown for the epididymis). Scale bar = 50 µm. (B) Semi-quantitative analysis of CD3+ cell foci in the MGT organs. The number of CD3+ cells in each focus was determined using the Cast software. The number of foci in each category (<50 cells, 50–250 cells, >250 cells) was counted on whole sections of seminal vesicle, prostate and epididymis from untreated macaques (n = 5) or treated for 2 (n = 3) or 4 weeks (n = 3). Stars indicate statistical differences (p<0.05) using a Mann-Whitney test. (C) Serial tissue sections were stained with anti-CD3 (a, a′), anti-TIA-1 (b, b′) and anti-CD4 (c, c′) antibodies. Photos are shown for the prostate (a, b, c) and seminal vesicle (a′, b′, c′) of one treated animal with undetectable PVL. Scale bar = 50 µm. (D) Quantitative analysis of CD68+ cells in seminal vesicle, prostate, epididymis and testis of untreated (n = 5) and treated macaques (n = 4).

In agreement with our published data [14], we evidenced the presence of HLA-DR+ immune cell infiltrates (mainly composed of CD3+ T lymphocytes) in all MGT organs apart from testis, from all the untreated macaques included in this study. In treated macaques, HLA-DR+ cell infiltrates were also encountered in the epididymis, prostate and seminal vesicle (Figure 3A). Similarly to untreated animals, these infiltrates were primarily composed of CD3+ T lymphocytes, and to a lesser extent of CD20+ B lymphocytes, whereas CD68+ myeloid cells were very rarely encountered (as shown for epididymis, Figure 3A). A semi-quantitative analysis revealed that in the seminal vesicle, the number of medium size CD3+ infiltrates was significantly lower in 4 week treated animals compared with untreated animals (Figure 3B). In the prostate, there was no significant difference between the numbers of infiltrates in the three groups of animals, but a trend for a lower number of medium/large sized infiltrates was observed in treated animals. In the epididymis of 2 and 4 week treated animals, medium size infiltrates were significantly lower when compared with untreated animals (Figure 3B). In both treated and untreated animals, the number of small, medium and large infiltrates was not significantly different between the different organs. In the accessory glands from untreated animals, all CD3+ T cell infiltrates were consistently composed of a mix of CD4+ T helper and TIA1+ cytotoxic cells (data not shown). In treated animals, most small/medium size T lymphocyte infiltrates in the epididymis, prostate and seminal vesicle were also encompassing a mix of CD4 and TIA1+ cells, although some infiltrates were predominantly composed of CD4+ cells (Figure 3 C). For all MGT organs, the number of CD68+ myeloid cells was in the same range for both treated and untreated macaques (Figure 3 D).

### Effect of short term HAART initiated 4 h post-inoculation

**Figure 4:**
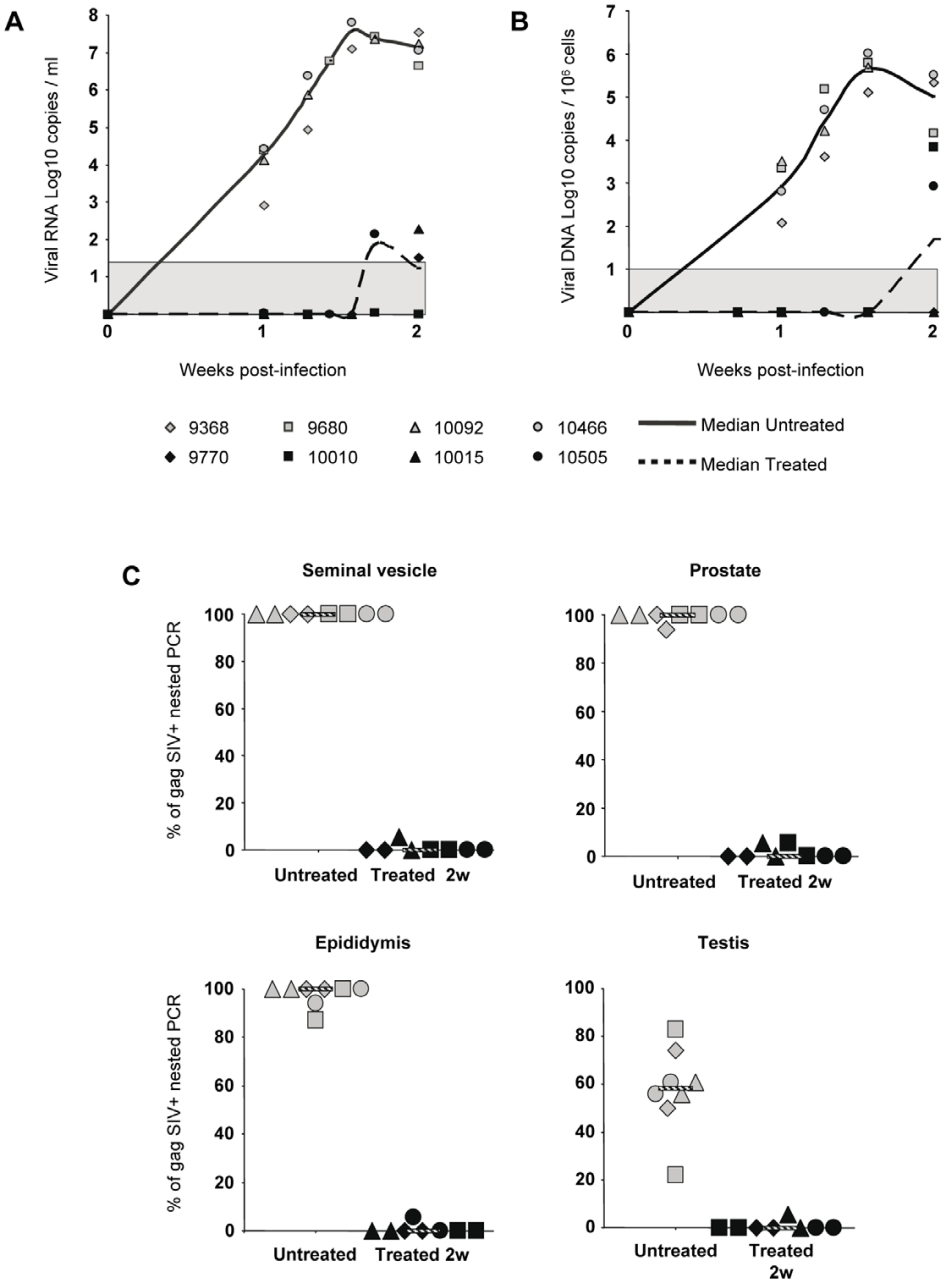
Viral dynamics in acutely-infected macaques receiving HAART and SIV nucleic acids detection in MGT organs. 4 macaques received a AZT/3TC/IDV combination (HAART 4 h-2 weeks) while 4 others were untreated. (A) Plasma viral load and (B) total viral DNA in PBMCs were measured during the 2 week protocol. Median is represented by a line. The grey area indicates the quantitative threshold of the qRT-PCR and qPCR assays. (C) Frequency of detection of SIV DNA in the seminal vesicle, prostate, epididymis and testis of untreated and 2 week treated macaques, using nested SIV gag PCR. Two independent fragments of each tissue were assayed. Median of frequency is represented by a line.

**Figure 5:**
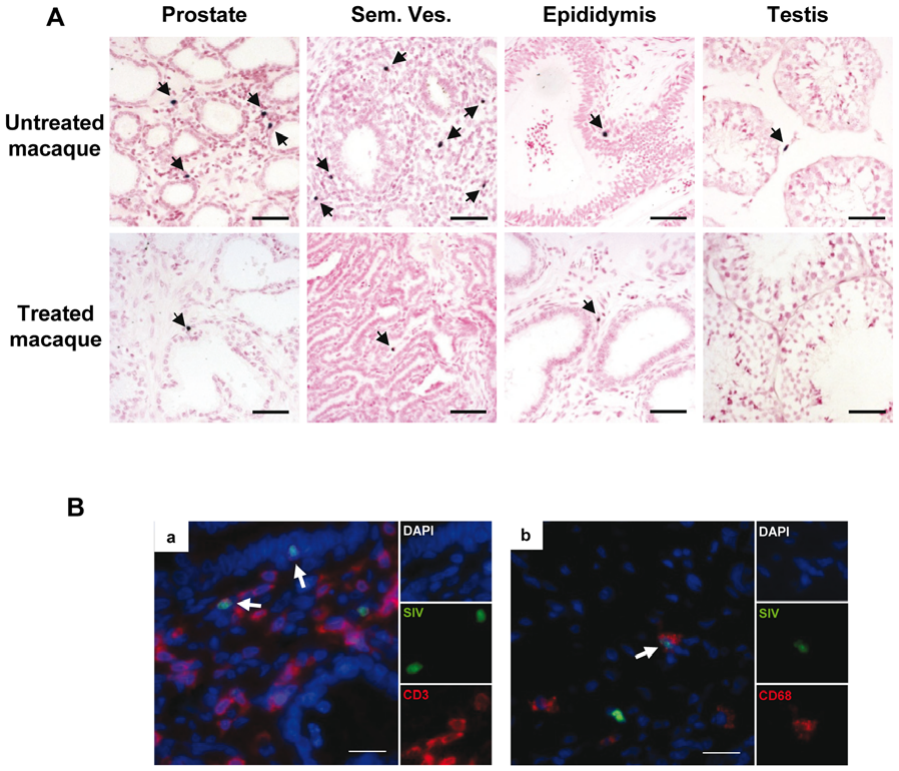
SIV RNA+ cells detection and identification within MGT of acutely infected macaques treated or not. (A) Non radioactive ISH for SIV viral RNA in the MGT organs of untreated versus treated animals. Arrows indicate SIV RNA+ cells. Scale bar = 50 µm. (B) Identification of SIV-expressing cells using non radioactive ISH for SIV (green) combined with immunofluorescence for cell marker CD3 (a, red) or CD68 (b, red). Nuclei labeled with DAPI are shown in blue. Side panels represent individual channels. Large panel represents a merged image combining all channels. Arrows show co-labeled cells. Scale bars = 20 µm.

**Figure 6:**
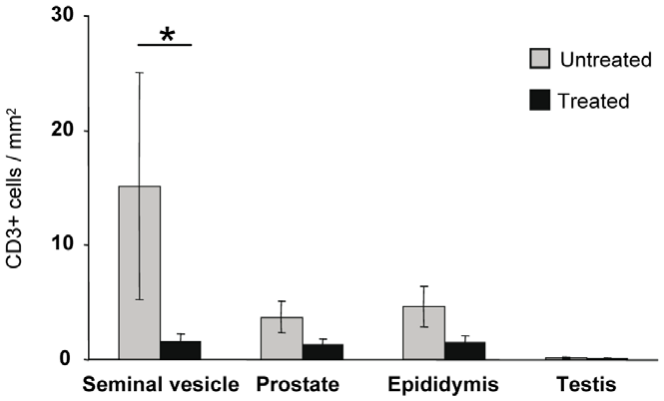
Quantitative analysis of CD3+ T lymphocytes in MGT organs of untreated and treated animals. Stars indicate statistical differences (p<0.05) using a Mann-Whitney test.

We previously showed that AZT/3TC/IDV combination initiated 4 h post-intravenous inoculation of SIV and maintained for 4 weeks does not prevent systemic infection, although in all treated animals it prevents the peak blood viral load [21]. As expected, although early treatment could not prevent infection, at week 2, a time we selected for necropsy around expected viremia peak, both PVL and PBMC viral load were either very low or at the limit of detection in treated animals (Figure 4 A, B). In contrast to our previous results on acutely infected animals without treatment [14], the level of SIV DNA in MGT organs of treated animals was too low to be measured by quantitative PCR. Nested PCR revealed that the level of SIV DNA was drastically reduced in all treated animals when compared with untreated animals (Figure 4 C). When detectable, the frequency of viral DNA detection was below 1% in all MGT organs of the treated animals, whereas it was between 80% to 100% for untreated animals, which was markedly higher than for chronically-infected animals (Figure 4 C versus Figure 1 C). Non radioactive ISH on untreated animals showed numerous SIV RNA+ cells in the prostate and seminal vesicle, and to a lesser extent in the epididymis and testis (Figure 5A), in agreement with our previous radioactive ISH quantitative results [14]. As previously described using a different technique [14], these infected cells were primarily CD3+T lymphocytes, as determined by combined non radioactive ISH for SIV RNA and immunostaining for cell markers (Figure 5B). Infected CD68+ macrophages were only occasionally encountered (Figure 5B). In treated animals, very scarce SIV RNA positive cells were detected in the prostate, seminal vesicle and epididymis and could not be evidenced in the testis (Figure 5A). We previously showed in untreated animals the presence of small CD3+T cell infiltrates in the prostate 2 weeks post-SIV inoculation [14]. Therefore, we assessed whether HAART affected the number of CD3+T lymphocytes count in MGT tissues. The median number of CD3+ T lymphocytes in the seminal vesicle of treated animals was significantly lower than in SIV+ untreated macaques (Figure 6). A similar trend was observed for the epididymis and prostate, although it did not reach significance (Figure 6).

## Discussion

This study is the first to investigate the effect of HAART on the infection of semen-producing organs during chronic or acute stage. A number of observations in HIV+ men (reviewed in [7], [31]) suggest that the male genital tract constitutes an HIV compartment distinct from blood, and a viral reservoir resistant to current antiretroviral therapies. The difficulty of access to semen-producing organs from healthy HIV+ men has prevented in depth investigations of these issues. Infection of macaques by SIV represents the best animal model of HIV infection. It is particularly useful for studying HIV pathogenesis, the effect of HAART in deep tissues, and acute stage of infection [21], [22], [32], [33]. We previously showed in this model that the seminal vesicle, the prostate, the epididymis and the testis are infected by SIV during the acute and chronic stage of the disease [14]. We confirmed the infection of these organs by HIV *in vitro* and *in vivo* [12], [13], [16], [17].

Persistent HIV shedding in semen has been shown to occur for a wide range of HAART regimens, including those based on the latest molecules, irrespective of the seminal concentrations achieved by the drugs [1], [3], [4], [5], [7], [31], [34], [35]. The AZT/3TC/IDV regimen has been associated in men with persistence of HIV RNA and DNA in semen despite prolonged undetectable blood viral load [4], [36], [37], [38], [39], [40] and good seminal concentrations of all three drugs [41].

Our results reveal that following 2 to 4 weeks administration of AZT/3TC/IDV during chronic stage, viral DNA and SIV RNA+ cells are still detected in the testis, epididymis, prostate and seminal vesicle of treated macaques with decreased systemic infection. Although precise quantification was hampered by the low level and focal nature of the infection, we did not observe any major impact of HAART on MGT organs infection. This contrast with our previous findings of a strong decrease of both viral DNA and RNA in the colon following the same HAART regimen and duration [22], which demonstrates the efficiency of this treatment in other body compartments. Similarly, 4 weeks of HAART has been shown to induce a rapid decline in RNA burden in blood, lymphoid tissues and brain of chronically infected macaques [42] and a 7 day treatment significantly decreased viral RNA in both blood and rectal mucosa in men [43]. In an analysis of potential body viral reservoirs following 26 weeks of HAART initiated 6 weeks post-infection, testis, prostate and seminal vesicle of RT-SHIV-infected juvenile macaques were also found to harbor viral DNA [44]. In contrast to our results, viral RNA was only detected in the prostate of one macaque out of five [44]. Limited sampling of the tissues and different detection techniques could have impaired detection of viral RNA in the MGT tissues in this study. Indeed we demonstrated the very focal nature of the infection and the need for testing multiple fragments. In addition, differences between this study and ours, including treatment duration and regimen, stage of infection, sexual maturity and virus used, could explain this discrepancy. Importantly, the presence of HIV p24 protein has been detected in the seminal vesicle of men receiving HAART [12].

The persistent detection of SIV nucleic acids in the genital organs of the treated macaques observed in our study could be due to either the existence of infected cells that would keep producing virus in the face of optimal drug concentrations during the 2–4 week period, or to low drug penetration in the organs. Unlike productively-infected CD4+T lymphocytes which are short-lived and usually rapidly eliminated following HAART [45], chronically infected tissue macrophages are generally regarded as long-lived reservoirs, in which RT inhibitors are ineffective and protease inhibitors display lower antiviral activity than in T lymphocytes [46], [47]. SIV/HIV infected macrophages have been previously detected in the testis, epididymis, prostate and seminal vesicle of macaques and men, along with infected CD4+ T lymphocytes [12], [13], [14], [15], [16], [48], [49], [50] and could thus contribute to the production of virus despite good drug concentrations in the organs. Using combined *in situ* hybridization for SIV and immunohistochemistry for cell markers, we confirmed the presence of infected macrophages and T lymphocytes in the MGT organs of untreated animals. In treated animals, both infected macrophages and lymphocytes were also detected. Despite our extensive screening of a large number of tissue sections, the relative proportion of infected macrophages and T lymphocytes could not be assessed, as accurate quantification was impaired by the low number of infected cells detected. However, the fact that productively infected CD3+ T cells were still detected at the end of the 4 week treatment period, and that the SIV DNA levels and SIV RNA+ cell number did not appear to be affected by the treatment, suggest that poor penetration of the antiretroviral drugs may also be a factor. Whether a longer and intensified treatment has more impact on male genital organs infection in our model will be tested in forthcoming experiments.

In men receiving HAART, drastic decreases in both blood and seminal viral loads are usually observed within one month post treatment initiation [51], [52], [53], and have been shown to occur as early as 14 days post HAART [3]. Even in persistent shedders, an initial decrease in semen viral load can be observed [3]. Our results suggest that this rapid decrease is unlikely to be due to a decrease in the infection of semen producing organs and most probably reflects the decrease of passive diffusion of HIV from blood into semen. Unfortunately, semen samples were not available at the time of our study to test this hypothesis. An ongoing study in our laboratory is currently exploring the impact of longer HAART on both semen and MGT organs infection.

Migration of T cells to non lymphoid tissues following virus-driven T lymphocytes expansion is a feature of chronic viral infection [54], [55]. In chronically-infected macaques with PVL>3 log10, we previously demonstrated in all MGT organs but the testis, the presence of HLA-DR+ immune cell infiltrates, mainly composed of cytotoxic and CD4+ T lymphocytes [14]. Here a lower number of CD3+ T cell infiltrates was observed in several MGT organs of treated *versus* untreated animals. This reduction did not appear to be linked to the organ infection levels, as the latter were not significantly different before/after treatment and between the organs. Therefore, we hypothesize that the reduced number of inflammatory infiltrates in MGT tissues may represent reduced systemic immune activation and T cell migration, in association with the lower blood viral loads in the treated animals [54], [56], [57], [58]. Alternatively, we cannot exclude that the treated macaques had lower level of inflammatory infiltrates before treatment compared with the untreated group. However, this seems unlikely as, apart from animal 20929, the viral load of treated animals before treatment was above 3 log10 and in the same range as the untreated animals.

We previously reported that 2 weeks post intravenous inoculation, SIV infected all MGT organs of cynomolgus macaques and that the viral load in MGT organs during the acute stage was much higher than during the chronic stage [14]. In this study we show that short term HAART initiated 4 h post-inoculation drastically decreased this infection to almost nothing, although some rare HIV-infected cells were still occasionally encountered. In treated animals, a significantly lower number of T lymphocytes were observed in the seminal vesicle as compared with untreated animals. A trend for a lower number of T lymphocytes was also observed for the epididymis and prostate of treated versus untreated animals. Intravenous inoculation of SIV has previously been reported to increase the number of T lymphocytes in several lymphoid and non lymphoid tissues 1 to 2 weeks post-infection [59], [60] and increased trafficking of T lymphocytes was evidenced in the gut as early as 48 h post-infection [61]. It is thus most likely that the post-exposure treatment reduced the number of infiltrating T lymphocytes in the MGT organs.

In humans and macaques infected through sexual routes, productive systemic infection begins around 10 days following amplification at transmission site and in local lymphoid tissues [62], [63]. Unlike our model in which viral dissemination occurs very quickly post inoculation [21], [64], this delay leaves a window of opportunity for preventing HIV systemic infection. Recommendations for post-exposure prophylaxis are to initiate HAART within 4 h (French guidelines) [65] to 3 days (US guidelines) [66]. Our results suggest that early HAART in men could significantly reduce MGT organs infection, even when systemic infection is not prevented. This could prevent the occurrence of persistent shedding in semen despite HAART, observed in some chronically infected individuals. However, the route of infection, i.e. intravenous, intrarectal or through foreskin/urethra, is likely to modify the outcome of early treatment aiming at preventing HIV dissemination. For instance, entry through the foreskin/urethra may result in HIV replication in the local lymph nodes draining MGT organs, and result in rapid infection of the latter. Further investigations will be needed to investigate this issue.

In conclusion, we showed for the first time that short term HAART has no major effect on the infection of MGT organs when initiated during the chronic stage, whereas post-exposure treatment drastically decreases their infection. These results indicate that persistent HIV shedding in men receiving HAART with undetectable blood viral load may arise from viral production in several semen producing organs. Our work opens several perspectives. Studies on longer treatment duration, intensification and measurement of antiretroviral drugs in male genital organs are now needed to assess whether the MGT constitutes a long term reservoir. In addition the comparison of viral load and strains in semen with that in male genital organs during HAART will be crucial to decipher further the origin of HIV persistence in semen. These issues are currently under investigation in our laboratory.
